# Very low prevalence of epidermal growth factor receptor (EGFR) protein expression and gene amplification in Saudi breast cancer patients

**DOI:** 10.1186/1746-1596-6-57

**Published:** 2011-06-24

**Authors:** Mohamed A Shawarby, Dalal M Al-Tamimi, Ayesha Ahmed

**Affiliations:** 1 Department of Pathology, College of Medicine, University of Dammam, Dammam, Saudi Arabia

## Abstract

**Background:**

Breast cancers which demonstrate EGFR protein expression, gene amplification and/or gene mutations may benefit therapeutically from tyrosine kinase inhibitors. In Western studies, EGFR protein expression has been demonstrated in 7-36% of breast cancer patients, while gene amplification has been found in around 6% of cases and mutations were either absent or extremely rare. Studies addressing EGFR protein expression and gene amplification in Saudi breast cancer patients are extremely scanty and the results reported have been mostly non-conclusive. Herein we report the prevalence of EGFR protein expression and gene amplification in a cohort of Saudi breast cancer patients.

**Findings:**

We noticed a remarkably low incidence of EGFR protein expression (1.3%) while analyzing the spectrum of molecular subtypes of breast cancer in a Saudi population by immunohistochemistry. Also, *EGFR *gene amplification could not be demonstrated in any of 231 cases studied using silver enhanced *in situ *hybridization.

**Conclusions:**

The extremely low incidence of EGFR protein expression and gene amplification in Saudi breast cancer patients as compared to Western populations is most probably ethnically related as supported by our previous finding in the same cohort of a spectrum of molecular breast cancer types that is unique to the Saudi population and in stark contrast with Western and other regionally based studies. Further support to this view is provided by earlier studies from Saudi Arabia that have similarly shown variability in molecular breast cancer subtype distribution between Saudi and Caucasian populations as well as a predominance of the high-grade pathway in breast cancer development in Middle East women. More studies on EGFR in breast cancer are needed from different regions of Saudi Arabia before our assumption can be confirmed, however.

## Findings

### Background and research hypothesis

EGFR is a tyrosine kinase receptor in the HER family which is widely expressed in a number of epithelial tumors and is believed to play a key role in cell proliferation. It is now well established that non-small-cell lung cancers which demonstrate EGFR protein expression, gene amplification and/or gene mutations at exons 18 - 21 show a dramatic therapeutic response to tyrosine kinase inhibitors such as gefitinib and erlotinib [1-3]. Although the same may be true for other cancers including breast cancer, data regarding the presence or absence of EGFR abnormalities in tumors other than lung cancer and the response of such tumors to anti EGFR therapy are still limited and rather conflicting. EGFR protein expression as assessed by immunohistochemistry has been demonstrated in 7-36% of breast cancer patients, while gene amplification as assessed by CISH or FISH has been found in around 6% of cases [4-8]. Mutations in exons 18 - 21 of the *EGFR *gene investigated by PCR were either absent [1,7] or present in only rare breast cancer patients [9], such mutations being much frequent in lung cancer [10]. Differences in the prevalence of EGFR over-expression reported by different studies have been attributed to probable variations in techniques and type of antibodies used, criteria for determining over-expression and inter-observer variability [7].

In a recent study that analyzed the spectrum of molecular subtypes of breast cancer in a Saudi population [11], we noticed (but have not reported) a remarkably low incidence of EGFR protein expression in our patients. Also, *EGFR *gene amplification could not be demonstrated in any of 231 cases studied using silver enhanced *in situ *hybridization (assessed after the study was published). In this article we aim to explore whether this extremely low incidence of protein expression and gene amplification reflects a truly low prevalence of *EGFR *gene abnormalities in the Saudi population which may be ethnically related or is, alternatively, due to possible suboptimal sensitivity of the immunohistochemistry technique/antibodies or the *in situ *hybridization method used.

### Patients, methods and results

We have recently published a study that analyzed the spectrum of molecular subtypes of breast cancer in 231 Saudi patients [11]. The age of the patients ranged between 25 and 97 years with a mean of 49.5 years (SD ± 11). Representative cancerous tissues obtained from paraffin blocks of mastectomy and lumpectomy specimens were incorporated into 5 tissue microarray reception blocks, from which 4 micron thick sections were cut for immunohistochemical and *in situ *hybridization studies. For tru-cut biopsies, conventional paraffin blocks were utilized. The cases were randomly selected from the archives of our pathology department based on the availability of representative blocks and sufficient tissue material to perform the required procedures. An immunohistochemical panel including ER, PR, HER2, Ck5/6 and EGFR antibodies was used as a surrogate for gene expression profiling to classify the 231 breast cancer specimens. Moreover, each class was correlated with its Ki-67 proliferation index and p53 gene over-expression, as revealed by IHC, and also with the histologic type and grade of the tumor. The histopathological and molecular charcteristics of breast cancer in these patients are shown in table 1.

**Table 1:** Histolopathogical and molecular characteristics of cancer in a cohort of 231 Saudi breast cancer patients

*Histologic type*	LUMA No.(%)	LUMB No(%)	HER2 No(%)	Basal No(%)	Hybrid No(%)	UC N0(%)	Total No(%)
*IDC*	6 (3.3)	27 (14.8)	29 (15.8)	20 (10.9)	16 (8.7)	85 (46.4)	183 (79.2)

*ILC*	3 (33.3)	5 (55.6)	0 (0)	0 (0)	0 (0)	1 (11.1)	9 (3.89)

*ISC*	0 (0)	3 (20.)	7 (46.7)	0 (0)	3 (20)	2 (13.3)	15 (6.49)

other*	0 (0)	2 (8.3)	4 (16.7)	3 (12.5)	4 (16.7)	11(45.8)	24 (10.38)

IDC = Invasive ductal carcinoma-NOS, ILC = Invasive lobular carcinoma, ISC = In situ carcinoma, LUMA = luminal A, LUMB = luminal B, HER2 = human epidermal growth factor receptor 2, UC = unclassified

*Include medullary carcinoma, mixed ductal and lobular carcinoma, metaplastic carcinoma, apocrine carcinoma and juvenile secretory carcinoma

**Figure 1:**
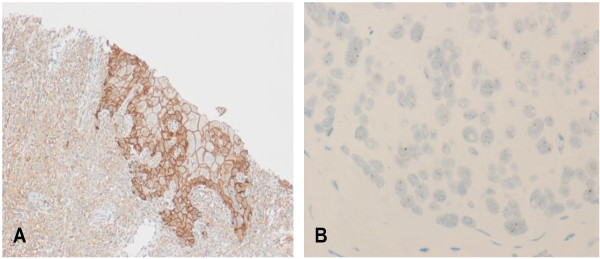
**EGFR protein expression by immunohistochemistry and gene amplification by SISH in a case of metaplastic breast carcinoma**. A) Membrane positivity by immunohistochemistry, × 100 B) No gene amplification (less than 5 gene copies per nucleus) by SISH, × 400.

The anti EGFR antibody was used solely as an indicator of the basal molecular subtype (together with CK5/6) and we have not reported or commented on the prevalence of EGFR protein expression among the studied cohort. A revisit to the study revealed that only three out of 231 cases were positive for EGFR (1.3%). Positivity was defined as membrane staining (Figure 1A) and was scored according to the criteria originally developed for HER2/neu into 0, 1+, 2+ and 3+ [7]. Only 2+ and 3+ membrane staining of 10% or more of the tumor cells was considered positive. Cytoplasmic staining alone was interpreted as negative. We used a primary antibody manufactured by Dako (clone H11 at a dilution of 1:200). The staining was performed in a Ventana Benchmark automated immunostainer according to the manufacturer's instructions (Ventana Medical Systems Inc., Tucson, Arizona). All three EGFR positive cases were negative for ER, PR and HER2 and two were also positive for CK5/6. We classified the three cases as "basal" based on Ck5/6 and/or EGFR positivity coupled with ER, PR and HER2 negativity. Table 2 shows the immunohistochemical findings in the EGFR positive cases including the Ki67 proliferation index which was high (70-100%). The patients were aged 35, 61 and 78 years. All had a high grade (grade III) invasive carcinoma but only one had an advanced (stage IV) disease (table 3). Although the number of the EGFR positive breast cancer cases is too small to allow for any correlation with clinical, pathologic or molecular variables, the presence of two "metaplastic" carcinomas out of three EGFR positive cases is in keeping with what has already been reported in the literature that approximately 70-80% of metaplastic breast carcinomas overexpress EGFR [12]. On the other hand, EGFR gene amplification - assessed after the study was published using the newly introduced silver enhanced *in situ *hybridization "SISH" technique (Ventana Medical Systems Inc., Tucson, Arizona) - could not be demonstrated in any of the 231 cases. The SISH detection kit utuilizes an enzyme labeled antibody that blocks the bound primary antibody. The complex is then visualized by silver acetate chromagen which produces a black precipitate. During the ISH process, labeled probes are bound to specific DNA or RNA target sequences in cells or tissues. Visualization of the bound linker antibody is accomplished through enzyme catalyzed deposition of silver. Silver ions are reduced by hydroquinone to metallic silver ions. The substrate for the enzyme catalyzed deposition of silver is hydrogen peroxide. The test was performed on 4 micron thick paraffin sections prepared from TMA and conventional blocks in a Ventana Benchmark IHC/ISH instrument (Ventana Medical Systems Inc., Tucson, Arizona) using an EGFR DNA probe (Ventana Medical Systems Inc., Tucson, Arizona) according to the manufacturer's instructions. The results were evaluated by light microscopy under a 40× objective. The SISH signals (black) were counted in at least 20 nuclei (Figure 1B). Gene amplification was defined as copy number greater than 5/nucleus.

**Table 2:** Immunohistochemical findings and Ki67 index in EGFR positive breast cancer cases

Case No	ER	PR	HER 2	CK 5/6	EGFR	Ki67 index
1	-ve	-ve	-ve	+ve	+ve (2+)	High (100%)

2	-ve	-ve	-ve	+ve	+ve (3+)	High (100%)

3	-ve	-ve	-ve	-ve	+ve (2+)	High (70%)

**Table 3:** Histologic type in relation to patient age, tumor grade and tumor stage in EGFR positive breast cancer cases

Case No	Histologic type	Patient age (years)	Tumor grade	Tumor stage
1	Metaplastic carcinoma	35	III	pT2N0M0 (IIA)

2	Invasive ductal carcinoma, NOS	61	III	pT2N0M0 (IIA)

3	Metaplastic carcinoma	78	III	pTXNXM1 (IV)

### Comment, conclusions and recommendation

The remarkably lower incidence of protein expression and gene amplification in our breast cancer cases as compared to that reported in Western studies (table 4) may reflect a truly low prevalence of *EGFR *gene abnormalities in the Saudi population which may be ethnically related. Alternatively, it may be due to possible suboptimal sensitivity of the immunohistochemistry technique/antibodies or the in situ hybridization method used. However, a much greater likelihood of some ethnic variation in *EGFR *gene abnormalities in breast cancer is supported by our previous finding in the same cohort of a spectrum of molecular breast cancer types that is unique to our population with luminal tumors comprising 19.9% and unclassified (penta negative) tumors 42.9% [11]. This distribution is in stark contrast with Western and other regionally based studies that have reported a prevalence of 44.5 - 80.2% for luminal cases [13-18] and 4.87 - 15.9% for the unclassified category [13-17]. Further support to this view is provided by earlier studies from Saudi Arabia that have similarly shown variability in molecular breast cancer subtype distribution between Saudi and Caucasian populations [19-21] as well as a predominance of the high-grade pathway in breast cancer development in Middle East women [19]. There are also other features that distinguish breast cancer in Saudi women from what is seen in Western populations. Breast cancers in Saudi women are generally locally advanced at the time of diagnosis, and affect predominantly females between 46-50 years of age, which is noticeably different from the median of 60-65 years seen in industrialized Western nations [22,23], where locally advanced disease is much less common.

**Table 4:** Prevalence of EGFR protein expression and gene amplification in present study compared to Western studies

	EGFR protein expression %	*EGFR *gene amplification %
Present study	1.3	0

Harris et al [4]	16	Not done

Tsutsui et al [5]	36	Not done

Walker & Dearing [6]	36	Not done

Bhargava et al [7]	7	6

Press et al [8]	27.9	Not done

A better approach to verify our assumption, however, would be to attempt confirming an extremely low prevalence of *EGFR *gene amplification in Saudi patients using PCR which is a more sensitive method than all in situ hybridization techniques. Moreover, by PCR, we can explore mutations at various exons of the *EGFR *gene, which may not necessarily be reflected as gene amplification or protein expression but are still effective in determining prognosis and response to anti EGFR therapy. Studies addressing EGFR protein expression and gene amplification in Saudi breast cancer patients are extremely scanty and the results reported have been mostly non-conclusive [19]. More studies in this direction are encouraged from different regions of Saudi Arabia.

## List of abbreviations

CK: Cytokeratin; CISH: Chromagenic *in situ *hybridization; EGFR: Epidermal growth factor receptor; ER: Estrogen receptor; FISH: Fluorescent *in situ *hybridization; HER: Human epidermal growth factor receptor; IHC: Immunohistochemistry; ISH: *In situ *hybridization; PCR: Polymerase chain reaction; PR: Progesterone receptor; SISH: Silver enhanced *in situ *hybridization;

## Competing interests

The authors declare that they have no competing interests.

## Authors' contributions

MAS and DMT were responsible for interpretation of IHC and SISH results. MS, DT and AA were involved in the writing up of the manuscript. All authors have read and approved the final manuscript.
